# Co‐Occurrence of Endogenous and Exogenous Cushing's Syndromes: Does “Double Cushing Syndrome” Really Exist? A Case Report

**DOI:** 10.1002/ccr3.71419

**Published:** 2025-11-10

**Authors:** Reza Amani‐Beni, Atiyeh Karimi Shervedani, Bahar Darouei, Matin Noroozi, Maryam Heidarpour

**Affiliations:** ^1^ Heart Failure Research Center, Cardiovascular Research Institute Isfahan University of Medical Sciences Isfahan Iran; ^2^ School of Medicine Isfahan University of Medical Sciences Isfahan Iran; ^3^ Isfahan Endocrine and Metabolism Research Center Isfahan University of Medical Sciences Isfahan Iran

**Keywords:** adrenocortical adenoma, case report, cushing syndrome, double cushing, glucocorticoids

## Abstract

Double Cushing syndrome exists: exogenous steroid use can mask concurrent adrenal hypercortisolism. When symptoms persist and cortisol remains high after tapering or stopping prescribed glucocorticoids, an endogenous source is likely. Early recognition with ACTH testing, dexamethasone suppression, and adrenal imaging reduces misdiagnosis, favors timely surgery, and supports safe tapering.

## 1 Introduction

Cushing syndrome (CS) is a non‐physiological increase in plasma glucocorticoids [1]. In most cases, the source of increased plasma glucocorticoids is caused by exogenous steroid administration, which is quite common, and about 1% of the world population is on long‐term (more than 3 months) oral glucocorticoids [1, 2]. On the contrary, endogenous overproduction of glucocorticoids is rare, and annually, only two to eight per million people are diagnosed with endogenous CS [3]. The simultaneous occurrence of endogenous and exogenous CS is an exceptionally uncommon phenomenon. This dual manifestation has been reported in a few case reports, highlighting its rarity and the complex diagnostic and therapeutic challenges it poses [4, 5]. Therefore, in this study, we discuss a patient who presented with cushingoid features and was simultaneously diagnosed with both endogenous and exogenous CS or, as it is called, double CS.

## 2 Case Presentation

The patient was a 46‐year‐old male with a history of new‐onset hypertension and recurrent deep vein thrombosis (DVT) who was referred to our endocrinology clinic with a chief complaint of hip pain and weakness of the lower limbs. In the past 3 years, the patient had been receiving 50 mg/day of oral prednisolone and inhalation powder of Umeclidinium and Vilanterol (62.5/25 μg/dose) because of respiratory complications that started after Coronavirus Disease 2019 (COVID‐19) vaccination. After 3 months of corticosteroid treatment, he experienced DVT for the first time when he was started on rivaroxaban. However, while he was on treatment, the second DVT occurred 1 month before his referral, and therefore, rivaroxaban was changed to warfarin 5 mg/day.

The patient also mentioned weight gain with his body mass index (BMI) rising from 26 to 31 kg/m^2^, progressive weakness of proximal muscles, easy bruising, decreased libido, mood changes with mostly euphoric mood, and irritability during the last 2 years. Moreover, multiple osteoporotic fractures of ribs, clavicle, sternum, and lumbar vertebrae were added to his symptoms in the past 5 months. At that time, he underwent bone densitometry, which revealed osteopenia of the left hip with a Z‐score of −1.3 and severe osteoporosis of total lumbar spine with a T‐score of −3.9. He started taking calcium and vitamin D3 supplements and received a single injection of 750 μg/3 mL teriparatide 30 days before his referral to our center.

Two months ago, the patient gradually reduced the dosage of prednisolone by tapering the dose to 12.5 mg/day. However, a month later, the hip pain and muscle weakness worsened to such an extent that the patient was unable to walk. Due to his signs and symptoms, the patient was referred to our center for further evaluation of CS. The patient also mentioned a history of nephrolithiasis, new‐onset hypertension, and lower limb edema, for which he was started on eplerenone 25 mg and furosemide 20 mg tablets once daily. In his family history, the patient's mother had type 2 diabetes mellitus, and his two sisters had a history of nephrolithiasis. The patient did not mention any history of allergies to medications or foods. He was addicted to opium and had 15 pack‐years of smoking, but he did not mention alcohol consumption.

**FIGURE 1:**
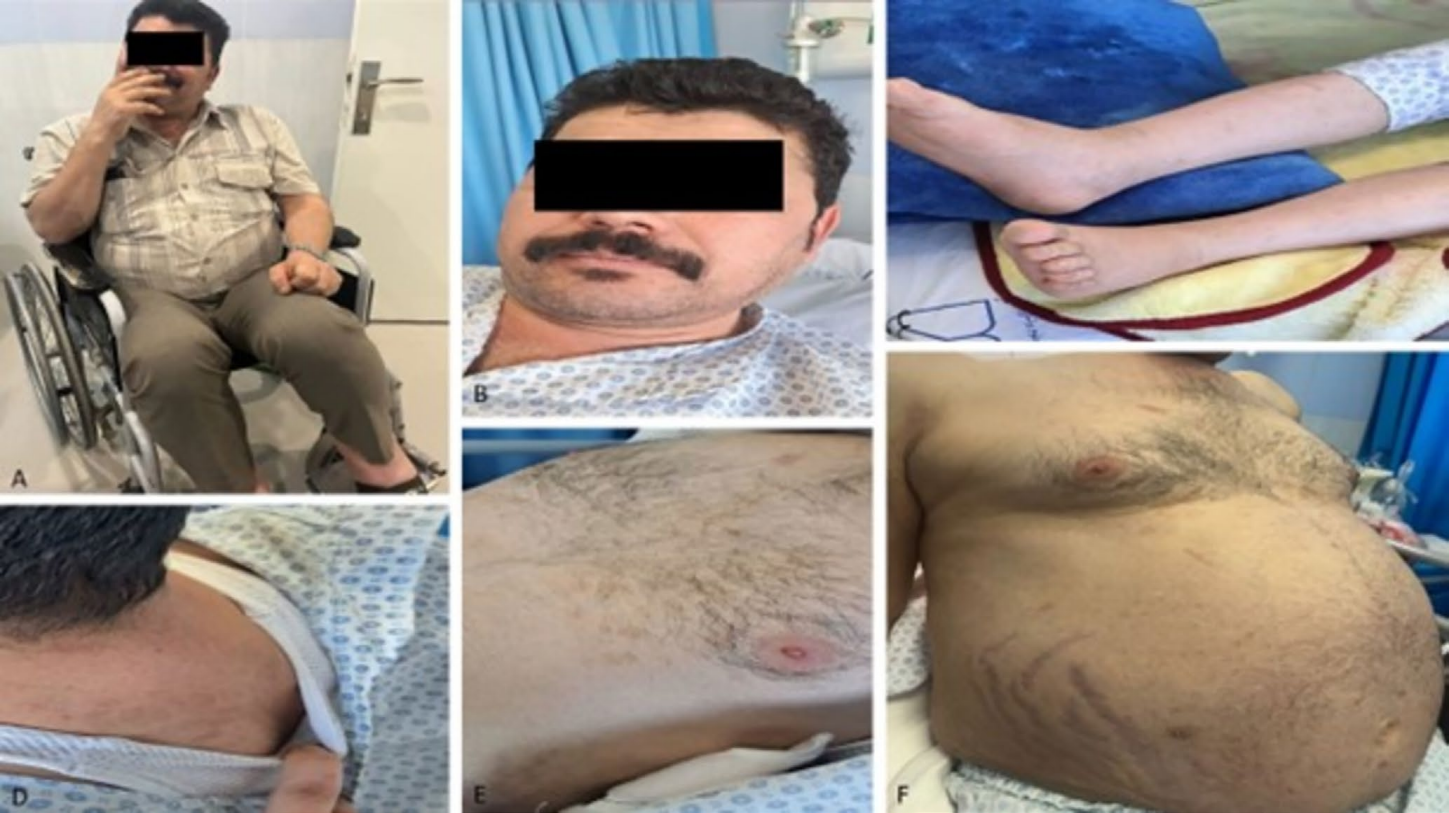
De‐identified clinical photographs illustrating the Cushingoid phenotype. (A) Overall habitus with marked central (truncal) adiposity. (B) Rounded plethoric face (“moon facies”). (C) Relatively slender distal extremities compared with truncal obesity. (D) Dorsocervical fat pad (“buffalo hump”). (E) Upper thoracic/supraclavicular fat accumulation. (F) Protuberant abdomen with wide violaceous striae.

Upon admission, the patient presented with a blood pressure of 150/83 mmHg, heart rate of 74 bpm, respiratory rate of 20/min, temperature of 36.5°C, oxygen saturation of 93%, and BMI of 31 kg/m^2^. He was sitting in a wheelchair due to weakness and severe pain in the hip. On physical examination, the patient showed the features of CS, including moon face, buffalo hump, central obesity, facial plethora, thin and brittle skin, acne, and purple stretch marks (striae) on the flanks (Figure 1). Proximal muscle weakness in the lower limbs with a muscle force grade of 4/5 and 3+ edema was also observed. Laboratory investigations are shown in Table 1.

**TABLE 1:** Laboratory findings of case report.

Laboratory test	Patient value (in‐hospital)	Patient value (follow‐up)	Reference range
On admission			
Hemoglobin (g/dL)	16.6	13.6	13.5–17.5
Hematocrit (%)	49.5	42.1	42–52
WBC (white blood cells; 10^3^/μL)	11.8	7.1	4.0–11.0
PLT (platelet count; 10^3^/μL)	286	294	150–450
BUN (blood urea nitrogen; mg/dL)	10	11	7–18
Cr (creatinine; mg/dL)	0.9	0.9	0.7–1.3
ALP (alkaline phosphatase; IU/L)	1016	129	44–147
AST (aspartate aminotransferase; IU/L)	48	30	< 31
ALT (alanine transaminase; IU/L)	88	21	< 31
CRP (C‐reactive protein; mg/dL)	31	3	< 5
ESR (erythrocyte sedimentation rate; mm/h)	63	24	< 15
Sodium (mEq/L)	148	141	136–145
Potassium (mEq/L)	4.8	4.3	3.5–5
FBS (fasting blood glucose; mg/dL)	97	89	80–100
TC (total cholesterol; mg/dL)	267	182	< 200
TG (triglyceride; mg/dL)	148	104	< 200
LDL (low‐density lipoprotein; mg/dL)	138	98	< 130
HDL (high‐density lipoprotein; mg/dL)	64	55	30–70
In hospital			
Cortisol 8 a.m. fasting (μg/dL)	20.2	14.1	4.3–24.9
ACTH (adrenocorticotropic hormone; pg/mL)	< 1	—	7.2–63.3
1 mg Overnight dexamethasone suppression test (μg/dL)	16.5	—	< 1.8

## 3 Methods (Differential Diagnosis, Investigations, and Treatment)

Initially suspected of having exogenous‐induced CS, the patient's prednisolone was on hold for 3 days. Cortisol 8 a.m. fasting level, measured with Electrochemiluminescence (ECL) and adrenocorticotropic hormone (ACTH) test, was 20.2 μg/dL (585.4 nmol/L) and < 1 pg/mL, respectively. Due to the lack of suppression of serum cortisol despite not using oral glucocorticoids, the absence of adrenal insufficiency symptoms, and the fact that the patient's symptoms remained unchanged during this period, co‐occurrence of endogenous CS was suspected.

**FIGURE 2:**
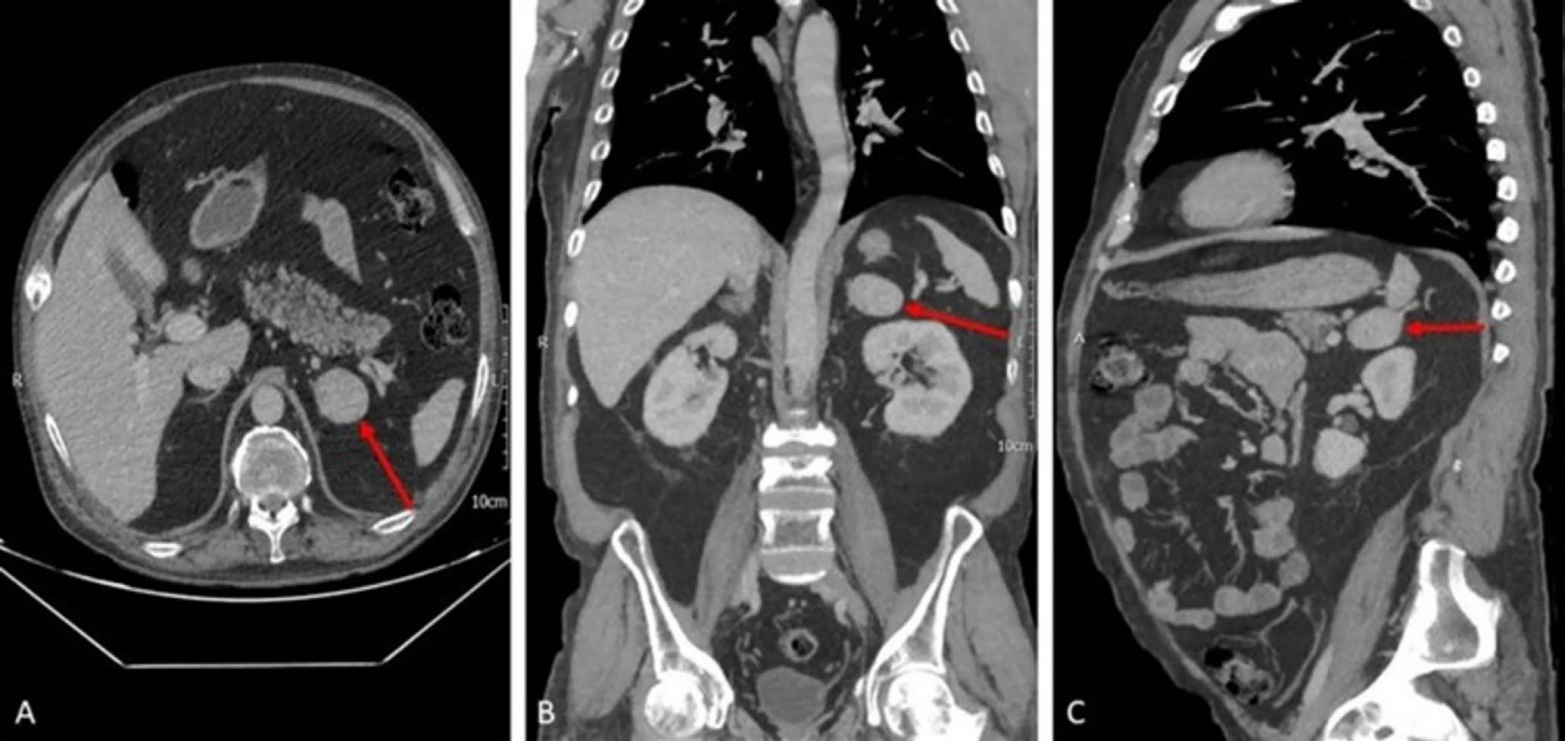
Abdominopelvic multidetector computed tomography (MDCT) with adrenal protocol showing a well‐defined lesion with an approximate size of 32.8 × 38.6 mm in the left adrenal gland; radiodensity 90 HU. (A) Transverse plane. (B) Coronal plane. (C) Sagittal plane.

A 1 mg overnight dexamethasone suppression test was performed to confirm endogenous CS diagnosis, and the results were reported as 16.5 μg/dL (normal range < 1.8 μg/dL). Considering the possibility of an ACTH‐independent CS, the patient underwent an abdominopelvic multidetector computed tomography (MDCT) of abdominopelvic with adrenal protocol, which revealed a well‐defined lesion with an approximate size of 32.8 × 38.6 mm in the left adrenal gland with a radiodensity of 90 Hounsfield units and a normal right adrenal gland (Figure 2). Moreover, evidence of previous old fractures as multiple callus formation was seen involving the clavicles, sternum, bilateral ribs, ischium, and pelvic bones. Multilevel old stable compression fractures of thoracic and lumbar vertebral bodies were also present. The differential diagnoses were glucocorticoid secretory adrenal tumors, including adrenal cell carcinoma and lipid‐poor adenoma. In order to rule out pheochromocytoma, 24‐h urine catecholamines were measured, and the results were negative.

**FIGURE 3:**
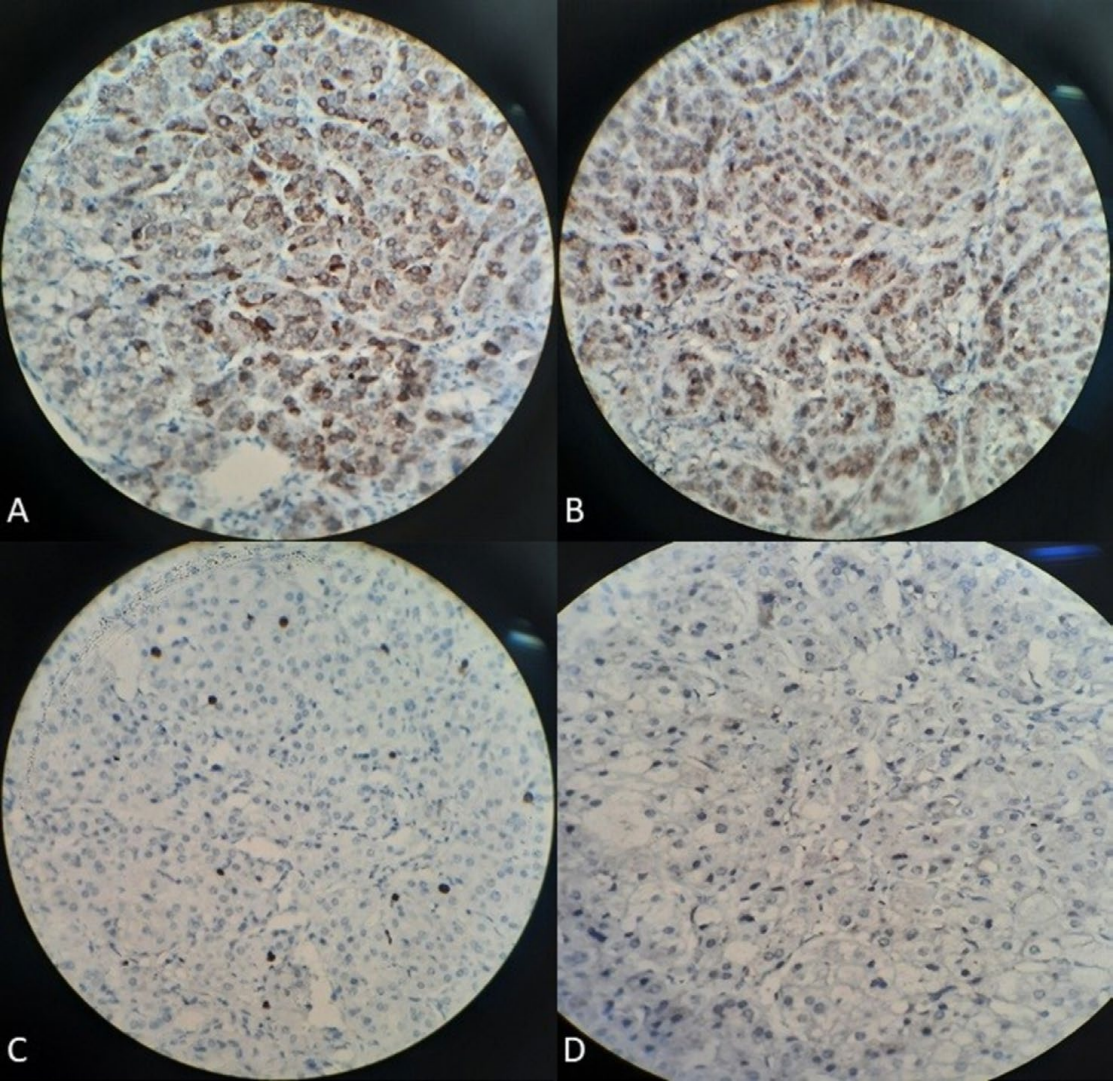
Immunohistochemistry of the adrenal lesion (all panels acquired with a 100× oil‐immersion objective; 10× eyepiece; original magnification ×1000). (A) Positive inhibin, (B) Positive Melan‐A, (C) Less than 3% Ki‐67 marker, and (D) Negative chromogranin.

Finally, the patient underwent left adrenalectomy and corticosteroid replacement therapy due to the suppression of the other adrenal gland. According to the post‐operative pathological investigations, immunohistochemistry markers reported as negative chromogranin, positive melan‐A and inhibin, less than 3% Ki‐67 marker, and lipid‐poor adrenal cortical adenoma without invasions were diagnosed (Figure 3).

## 4 Results (Outcome and Follow‐Up)

Within 3 months after the operation, the patient's corticosteroid was tapered and then discontinued due to the normalization of the cortisone serum test (14.1 μg/dL). Proximal limb weakness and hip pain, which had deprived the patient of the ability to move, gradually improved so that he could walk easily and perform daily activities. The signs and symptoms related to CS, including the patient's mood, skin signs, and general appearance, returned to normal. The patient has been followed up for 6 months after the surgery. The patient's BMI decreased to 24 kg/m^2^, and he stopped his anti‐hypertensive medications with a blood pressure of 100/60 mmHg without previously prescribed drugs. So far, the laboratory tests have been within the normal range, and he has no complaints (Table 1).

## 5 Discussion

The described case was diagnosed with a cortisol‐producing adrenocortical adenoma accompanied by exogenous CS. CS is an uncommon clinical condition caused by prolonged exposure to increased cortisol levels, which can be due to endogenous or exogenous factors [6]. Endogenous CS is infrequent and is classified as ACTH‐dependent (80% of cases) or ACTH‐independent (20% of cases) [7]. In the ACTH‐independent category, adrenal adenoma accounts for 60% of cases and only 12% of cases of endogenous CS [7, 8]. Exogenous CS mainly occurs due to prolonged administration of glucocorticoids, which are used to manage a broad spectrum of diseases such as inflammatory, autoimmune, or neoplastic disorders and are the most common cause of CS worldwide [9]. Multiple factors, including formulation, duration of administration, pharmacokinetics, affinity, and potency of exogenous glucocorticoids, affect the probability of exogenous CS, but all forms of glucocorticoids can induce CS [10].

In the setting of cushingoid clinical features with chronic administration of high‐dose glucocorticoids, especially oral prednisolone, the probability of exogenous CS is remarkably high; therefore, CS diagnostic approaches suggest that the first step after confirmation of cortisol excess is ruling out exogenous glucocorticoid administration [7, 8, 10]. Therefore, the possibility of co‐occurrence of endogenous CS with iatrogenic CS is extremely low, and the diagnosis requires high clinical suspicion [4].

Differentiating endogenous and exogenous CS based on clinical features can be challenging and far‐fetched. However, a few points can help physicians distinguish between these two. First, exogenous CS symptoms tend to be more striking, while endogenous CS appears more gradually. Second, hypertension, hypokalemia, and features of androgen excess, such as acne and hirsutism, are more common in endogenous CS [4, 10]. In addition, endogenous CS should be suspected if the patient's symptoms continue after corticosteroid discontinuation or if the serum cortisol level is high despite corticosteroid cessation. In our case, the patient had a high cortisol level despite stopping prednisolone for 3 days, and he did not have any symptoms of adrenal insufficiency despite stopping prednisolone suddenly. Consequently, it was suspected that glucocorticoids might come from an endogenous source. Because ACTH was suppressed concurrently with elevated cortisol, non‐ACTH‐dependent CS was suspected, and MDCT of abdominopelvic confirmed it.

So far, few similar cases of simultaneous endogenous and exogenous CS have been reported. The first case was a 23‐year‐old woman with juvenile idiopathic arthritis who was administered high doses of triamcinolone for 16 years [4]. The development of cushingoid features that favored endogenous CS, such as hirsutism and acne, strengthened the suspicion of endogenous CS, and a CT scan revealed hypercortisolism with a bulky and nodular left adrenal gland, and a double CS was confirmed [4]. The second case was a 66‐year‐old woman diagnosed with exogenous CS after consumption of Traditional Chinese medicine (TCM) for a year [5]. The cessation of TCM did not significantly improve her cushingoid features, and she developed additional CS complications, including hypertension, diabetes mellitus, and osteoporotic fractures over the next 8 years. CS workup revealed a right‐sided adrenal adenoma, and after the adrenalectomy, her clinical cushingoid features markedly improved [5]. These cases suggest that exogenous and endogenous CS can exist simultaneously in the same person. Although it is very rare, it should be considered in a person who still complains of CS symptoms after corticosteroid cessation. We suggest clinicians evaluate the patients for the disappearance of exogenous CS symptoms after tapering and stopping glucocorticoids. If the symptoms remain, they should be evaluated for endogenous CS.

## 6 Conclusion

The co‐occurrence of an endogenous CS in the setting of an exogenous CS is curious. The diagnosis is based on a high clinical suspicion. Clinicians should evaluate patients for symptom resolution after tapering and discontinuing corticosteroids. Clinical cushingoid features that do not resolve after discontinuing exogenous glucocorticoids and high cortisol levels despite discontinuing corticosteroids should raise clinicians' suspicion of the co‐occurrence of exogenous and endogenous CS.

## Author Contributions

**Reza Amani‐Beni:** investigation, methodology, writing – original draft, writing – review and editing. **Atiyeh Karimi Shervedani:** methodology, writing – original draft. **Bahar Darouei:** conceptualization, validation, writing – review and editing. **Matin Noroozi:** methodology, writing – original draft. **Maryam Heidarpour:** conceptualization, supervision, validation, writing – review and editing.

## Consent

Written informed consent was obtained from the patient to publish this report, including de‐identified clinical photographs, in accordance with the journal's patient consent policy.

## Conflicts of Interest

The authors declare no conflicts of interest.

## Data Availability

The data that supports the findings of this study are available on request of the corresponding author. The data are not publicly available due to privacy restrictions.
